# Technology Acceptance and Information System Success of a Mobile Electronic Platform for Nonphysician Clinical Students in Zambia: Prospective, Nonrandomized Intervention Study

**DOI:** 10.2196/14748

**Published:** 2019-10-09

**Authors:** Sandra Barteit, Florian Neuhann, Till Bärnighausen, Annel Bowa, Sigrid Wolter, Hinson Siabwanta, Albrecht Jahn

**Affiliations:** 1 Heidelberg Institute of Global Health Heidelberg Germany; 2 Department of Global Health and Population Harvard T.H. Chan School of Public Health Boston, MA United States; 3 Africa Health Research Institute (AHRI) KwaZulu-Natal South Africa; 4 Lewy Mwanawasa University Lusaka Zambia; 5 SolidarMed Lusaka Zambia

**Keywords:** computers, handheld, tablets, education, medical, sub-Saharan Africa, Zambia, mHealth, evidence-based practice, medicine

## Abstract

**Background:**

Zambia is still experiencing a severe shortage of health workers, which is impacting the national health care system. Very few people are trained, educational infrastructure is inadequate, and senior human resources for training are not yet sufficient to produce the number of health care workers needed, especially for currently underserved rural areas. Therefore, to strengthen the medical education program of medical licentiates, we implemented a tablet-based electronic learning platform (e-platform) with a medical decision-support component.

**Objective:**

As the primary objective, this study aimed to explore the acceptance and information system (IS) success of an e-platform focused on offline-based tablet usage for nonphysician clinical students in a low-resource context in Zambia, Africa. Furthermore, we aimed to evaluate student demographic factors and prior technological experience, as well as medical lecturers’ acceptance of technology of the e-platform.

**Methods:**

We collected data for the study before and after the intervention. Before the intervention, we collected student demographic data and prior technological experience using a questionnaire. After the intervention, we collected results of the questionnaire on technology acceptance of students and IS success of the e-platform, as well as technology acceptance of medical lecturers. We calculated statistical measures such as means, standard deviations, and correlations of investigated variables. The study report was compiled using the Consolidated Standards of Reporting Trials-Electronic Health checklist.

**Results:**

Overall, questionnaire results of students and medical lecturers indicated acceptance of the e-platform and showed higher ratings for overall net benefits and information quality (students) and perceived ease of use and perceived usefulness (medical lecturers) as compared with ratings of other categories. The lowest scores were conveyed for system use and service quality (students) and attitude and behavioral intention (medical lecturers).

**Conclusions:**

Acceptance of the e-platform as a learning technology for strengthening medical education in a low-resource context in Zambia was generally high for students and medical lecturers, but shortcomings were also identified. Results indicated low overall usage of the e-platform as a learning and teaching tool. One hindering factor was the tablets’ overall weak reliability with regard to its service life and battery life span, and another was the teachers’ low engagement with the e-platform. Next steps may include other hardware and more technology-based training for medical lecturers. The evaluation results indicated that the e-platform may open new promise for further strengthening and expanding medical education in this context, especially with more affordable and viable technologies that are available.

## Introduction

### Background

Zambia, a country in south-central Africa, faces significant challenges in its overall disease burden and scant numbers of health workers across all sectors and rural regions in particular [1-3]. The current severe shortage of Zambian health workers and their skewed distribution toward urban areas underline the dire need for an upscale of skilled and knowledgeable health workers, especially in rural areas. The ratio of health workers in Zambia compared with the population is 1.2 per 1000 people, which is far from the World Health Organization Sustainable Development Goals index threshold of 4.45 health workers per 1000 people [2,4]. In a country with over 750,000 km^2^ area, Zambia is sparsely populated by approximately 20 inhabitants per square kilometer [5], which further aggravates the dense coverage of and proximate access to health care. Zambia’s rural areas are severely underserved with only 7 clinicians per 10,000 people (urban areas: 16/10,000 people) and are insufficiently covered by health facilities. The recent Zambian National Health Strategic Plan [1] points out that at times “the situation is so severe that some facilities in rural areas have insignificant numbers of staff and in the worst scenario are managed by unqualified staff.” In 2002, to address the need for better-qualified nonphysician clinicians to cover a broader scope of health care than that provided by clinical officers, Zambia started training associate physicians at the Chainama College of Health Sciences (CCHS), Lusaka, to be known as medical licentiate practitioners (MLPs) [6]. MLPs manage advanced and common medical conditions and cover competencies such as surgical care, cesarean sections, and management of complex treatments. Most MLPs work at rural district hospitals or peripheral health centers. Retention of MLPs is high, and over 270 MLPs work in the Zambian health system, thus strengthening health care in rural areas [1]. As a result, the Ministry of Health has set an objective to more than double the MLP workforce to 600 by 2025 [1]. Although the number of MLPs trained at CCHS has increased, especially in rural areas, CCHS as the main national training institution for MLPs has experienced restricted resources and infrastructure to adequately upscale the sorely needed increase in the number of students.

### Research Objectives

To strengthen the quantity and quality of training for MLPs, we introduced a blended learning approach that includes a self-directed electronic health platform (e-platform) [7]. The primary objective of the e-platform is to alleviate the shortcomings of the medical licentiate program training caused by shortages of medical lecturers and learning materials in MLP training sites. The e-platform covers 2 components: (1) electronic learning (e-learning) for medical education with Web-based and offline static and interactive learning materials including lecture presentations, books, virtual patient cases, pictures, and videos and (2) health care practice support with medical treatment guidelines and algorithms to diagnose and treat patients. Details on e-platform contents are described elsewhere [8,9]. MLP students were provided with 7-inch tablets preloaded with offline contents of the e-platform through the Moodle mobile app [10]. The blended learning approach was piloted from January 2016 until August 2016 [8], and the evaluation of the first year of full implementation of the e-platform for the MLP was conducted from September 2016 to August 2017. The evaluation included both a qualitative [8,9] and quantitative methodology. The objective of the quantitative evaluation was to empirically explore the acceptance based on the technology acceptance model (TAM; TAM2 as a baseline model [11-13]) and information system (IS) success based on the IS success model (ISSM) [14] of the e-platform. The TAM assumes that the users’ acceptance of technology depends on 2 variables: (1) perceived usefulness and (2) perceived ease of use [15]. In the original TAM, these 2 variables served as a proxy to determine the users’ attitude toward using the technology [16]. In 2000, the extended TAM was proposed (TAM2) [17]to include social and cognitive influences to better reflect the complexity of the users’ technology acceptance decisions and the increased complexity of more developed ISs [15]. The ISSM is a measure for IS that is organized in 3 levels: (1) the first level includes ISSM quality constructs that impact the user’s satisfaction and intention to use the IS, (2) the second level examines the systems’ performance, and (3) the third level looks at net benefits of an IS.

With questionnaires based on the TAM2 and ISSM, we wanted to measure technology acceptance and IS success, respectively, answering the following specific research questions:

1. Is the technology of e-learning for medical education accepted, that is, do students and medical lecturers agree with the technological environment of e-learning as a mode of learning and teaching for the CCHS?
2. To which extent is medical e-learning used for learning and teaching by MLP students and medical lecturers and how is medical e-learning used Web-based and offline (context, frequency, materials and IS success respectively)?

## Methods

### Study Participants

During the study period from September 2016 to August 2017, a total of 83 students (3rd study year [SY] n=23, 4th SY n=32, and bridging SY n=28) were registered in the MLP at CCHS and together with the medical lecturers (n=36) constituted the total study population. All enrolled MLP students were eligible for the study, so no sample size specification was applicable. At the time of the study, the MLP student population included 3rd and 4th SY students and bridging SY students (those who had already acquired an MLP diploma in the past and continued 1 more year in the MLP to receive a Bachelor of Science degree).

### Study Design and Procedures

The prospective, nonrandomized intervention study was conducted during the MLP SY 2016/17 that started in September 2016 and ended in July 2017. The study took place in the capital—Lusaka—and 10 rural hospitals were used as practical training sites for the MLP. The CCHS main campus, a medical multiprofessional training institution, was located about 10 km east of Lusaka’s city center and was one of the largest training institutions for Zambian health workers.

#### Learning Materials

Learning materials for the e-platform mainly comprised readily available materials provided by medical lecturers according to the MLP curriculum and materials customized for the e-platform. Access to e-platform materials was available offline via the tablets and Web-based, via the Moodle learning management system [10]. E-platform contents included static and interactive elements, such as lecture presentations (mostly Microsoft PowerPoint), medical books, interactive virtual patient cases, medical images, and short videos on medical procedures (see Table 1). Health care practice was supported with materials on standard treatment guidelines and algorithms for diagnosis and treatment.

#### Data Collection

Fieldwork for this study was conducted in collaboration with CCHS. In September 2016, study baseline data were collected before the intervention and included student demographic data and technology experience. Students’ name, date, place, date of birth, marital status, SY, prior studies, year of graduation, and medical experience in years were collected (see Table 2) together with a 10-item paper-based questionnaire about technology experience (see Multimedia Appendix 1). A questionnaire asked students about their usage and exposure to computers and mobile computing devices, level of comfort of internet navigation, and prior experience with and self-perceived usefulness of e-learning.

After completion of the baseline data, the MLP students were given tablets (7-inch and Android-based) preloaded with learning and clinical decision-making support materials and access to the Web-based e-platform. MLP lecturers were provided access to the Web-based MLP e-platform.

In August 2017, students’ data were collected on technology acceptance and IS success with paper-based questionnaires (5-item Likert scale: strongly disagree, disagree, neutral, agree, and strongly agree). The students’ technology acceptance questionnaire comprised 25 questions based on the TAM2 baseline model [11-13] with 6 constructs (see Table 3): (1) perceived ease of use, (2) perceived usefulness, (3) attitude, (4) behavioral intention, (5) self-efficacy, and (6) subjective norm; and 55 questions comprised DeLone and McLean’s IS success model [14] on the basis of 6 constructs (see Table 4): (1) information quality, (2) service quality, (3) system quality, (4) user satisfaction, (5) system use (the intention to use the system), and (6) net benefits. MLP students completed the questionnaires in small groups of approximately 30 students. The TAM-based questionnaire for medical lecturers included 45 questions. Medical lecturers were invited to complete the questionnaire via a cloud-based survey service within a 3-month time frame (July to October 2017), as they were dispersed throughout Zambia.

This study report conforms with the Consolidated Standards of Reporting Trials-Electronic Health [18] checklist (see Multimedia Appendix 2).

**Table 1:** Available Zambian medical licentiate program learning materials on the electronic platform by medical specialty and content type.

Content type	Medical specialty				Total
	Internal medicine	Surgery	Obstetrics and gynecology	Pediatrics	
Lecture notes	38	64	43	31	176
Medical books	6	1	1	7	15
Exam preparation	1	0	0	1	2
Treatment guidelines	69	0	1	32	102
Videos	2	44	42	0	88
Pictures	0	0	0	39	39
Virtual patients	4	1	0	2	7

**Table 2:** Demographic data (summary statistics) of the study sample of medical licentiate practitioner (MLP) students and lecturers.

Variables			n (%)	Minimum	Maximum	Median	Mean (SD)
**MLP students (N=74)**							
	**Gender**			—^a^	—	—	—
		Female	16 (22)				
		Male	58 (78)				
	**Age (years)**			**23**	**54**	**36**	**38 (7)**
		≤30	10 (14)				
		>30-≤40	42 (57)				
		>40	22 (30)				
	**Medical experience (years)**			**1**	**28**	**9**	**10 (6)**
		≤5	21 (28)				
		>5-≤15	35 (47)				
		>15	18 (24)				
	**Technology experience score between 0 (minimum) and 1 (maximum)**			**0.06**	**0.88**	**0.69**	**0.67 (0.16)**
		Low	27 (36)				
		Moderate	36 (49)				
		High	11 (15)				
	**Study year**			—	—	—	—
		Third	20 (27)				
		Fourth	29 (39)				
		Bridging	25 (34)				
**MLP medical lecturers (N=14)**							
	**Gender**			—	—	—	—
		Female	4 (29)				
		Male	10 (71)				
	**Age (years)**			**38**	**54**	—	**45 (6)**
		≤30	0 (0)				
		>30-≤40	3 (21)				
		>40	11 (79)				
	**Medical experience (years)**			**9**	**27**	**14.5**	**17 (6)**
		≤5	0 (0)				
		>5-≤15	8 (57)				
		>16	6 (43)				
	**Teaching experience (years)**			**2**	**2**	—	**10 (6)**
		≤5	4 (29)				
		>5-≤15	8 (57)				
		>15	2 (14)				

^a^Not applicable.

**Table 3:** Questionnaire results of technology assessment model questionnaires of medical licentiate practitioner students.

Questionnaire items of technology acceptance component: MLP^a^ students		Mean (SD)	*r* ^b^
**Perceived ease of use**		1.82 (0.61)	0.61
	I find the MLP e-learning platform easy to use.	1.89 (0.57)	0.45
	Learning how to use the MLP e-learning platform is easy for me.	1.96 (0.60)	0.57
	It is easy to become skillful at using the MLP e-learning platform.	1.90 (0.64)	0.63
	Learning to operate my tablet is easy for me.	1.80 (0.71)	0.61
	I am clear on how to use the tablet.	1.77 (0.64)	0.65
	It is easy for me to become skillful at using my tablet.	1.68 (0.53)	0.69
	I find my tablet easy to use.	1.73 (0.51)	0.65
**Perceived usefulness**		1.85 (0.61)	0.60
	E-learning improves my learning performance.	1.93 (0.49)	0.49
	E-learning makes it easier to study course content.	1.96 (0.62)	0.49
	Using a tablet computer is compatible with all aspects of my studies.	2.04 (0.78)	0.64
	I think that using a tablet fits well with the way I like to learn.	1.90 (0.61)	0.73
	Using a tablet fits into my learning style.	1.83 (0.56)	0.66
	In my job, using a tablet is important.	1.61 (0.52)	0.64
	In my job, using e-learning is important.	1.65 (0.54)	0.50
**Attitude**		1.72 (0.59)	0.70
	Studying through e-learning is a good idea.	1.79 (0.59)	0.73
	Studying through e-learning is a wise idea.	1.76 (0.60)	0.75
	I am positive towards e-learning.	1.74 (0.61)	0.72
	I am positive towards using a tablet for medical learning.	1.60 (0.57)	0.59
**Behavioral intention**		1.69 (0.58)	0.67
	I intend to be a heavy user of the MLP e-learning platform.	1.80 (0.63)	0.55
	For my future job, it is necessary to know how to use a tablet.	1.67 (0.56)	0.69
	For my future job, it is necessary to know how to use a computer.	1.59 (0.55)	0.77
**Self-efficacy**		1.86 (0.69)	0.65
	I feel confident finding information on the MLP e-learning platform.	1.87 (0.63)	0.72
	I have the necessary skills for using the MLP e-learning platform.	1.94 (0.67)	0.65
	I feel confident using the MLP e-learning (Moodle app) with the tablet.	1.77 (0.76)	0.59
**Subjective norm**		1.92 (0.61)	0.78
	What e-learning stands for is important for me as an MLP student.	1.90 (0.61)	0.75
	It is necessary to take e-learning courses to train as an MLP.	1.93 (0.61)	0.80
	Information system success (for details see information system success model results)	2.13 (0.76)	0.29

^a^MLP: medical licentiate practitioner.

^b^*r*: corrected item-total correlations.

**Table 4:** Questionnaire results of information system success of medical licentiate practitioner students.

Questionnaire information system success component: MLP^a^ students		Mean (SD)	*r* ^b^
**Information quality**		1.99 (0.80)	0.57
	The MLP e-learning provides up-to-date learning materials.	2.06 (0.86)	0.58
	Learning materials available on the MLP e-learning platform are clear.	1.88 (0.72)	0.59
	Materials available on the MLP e-learning platform are useful to me.	1.58 (0.56)	0.49
	Materials available on the MLP e-learning platform help me to understand medical topics better.	1.89 (0.69)	0.57
	The quality of materials available on the MLP e-learning platform is high.	2.34 (0.85)	0.66
	The MLP e-learning provides information relevant to MLP medical practice.	1.63 (0.55)	0.56
	Materials on the MLP e-learning platform increase my quality of clinical care.	1.74 (0.51)	0.57
	The MLP e-learning platform provides sufficient learning materials.	2.51 (0.94)	0.65
	Through the MLP e-learning platform. I get access to learning materials I need in time.	2.29 (0.88)	0.48
**Service quality**		2.29 (0.86)	0.52
	The MLP e-learning platform provides proper level of assistance and explanation.	2.30 (0.74)	0.37
	The MLP e-learning platform was available when I wanted to access it.	2.65 (1.00)	0.33
	The local IT^c^ has adequate knowledge to help me if I experience any problems with the MLP e-learning platform.	2.28 (0.88)	0.66
	The local IT has adequate knowledge to help me if I experience any problems with the tablet.	2.07 (0.74)	0.62
	The IT provides satisfactory support to users of the MLP e-learning platform.	2.11 (0.87)	0.63
	The IT attends to my problems.	2.15 (0.84)	0.61
	The MLP e-learning platform provides dependable services.	2.24 (0.73)	0.52
	The MLP e-learning platform provides rapid services.	2.54 (0.92)	0.38
**System quality**		2.10 (0.70)	0.61
	The MLP e-learning platform is easy to use.	2.02 (0.77)	0.66
	The MLP e-learning platform is user-friendly.	1.94 (0.72)	0.69
	The MLP e-learning platform is easy to learn.	1.89 (0.61)	0.58
	Most MLP students find the MLP e-learning platform easy to use.	2.15 (0.66)	0.66
	Most MLP students find the MLP e-learning platform user-friendly.	2.23 (0.74)	0.65
	Most MLP students find the MLP e-learning platform easy to learn.	2.14 (0.65)	0.61
	The user interface of the MLP e-learning is attractive.	2.17 (0.69)	0.49
	The MLP e-learning platform has attractive features to appeal to users.	2.23 (0.72)	0.56
**User satisfaction**		2.11 (0.72)	0.62
	I think that most MLP students bring a positive attitude towards the MLP e-learning platform.	2.11 (0.61)	0.51
	I think that most MLP students have a high perceived utility about the MLP e-learning platform.	2.22 (0.63)	0.64
	I am satisfied with efficiency of the MLP e-learning platform.	2.30 (0.88)	0.70
	I will continue to use the MLP e-learning platform.	1.79 (0.67)	0.59
	Overall, I am very satisfied with the MLP e-learning platform.	2.14 (0.67)	0.66
**System use**		2.47 (0.84)	0.69
	My frequency of using the MLP e-learning platform is high.	2.33 (0.75)	0.67
	I use the MLP e-learning platform daily several times.	2.61 (0.90)	0.76
	I depend upon the MLP e-learning platform.	2.53 (0.82)	0.72
	I use the Moodle app daily several times.	2.40 (0.67)	0.60
**Net benefits**		2.00 (0.61)	0.60
	The MLP e-learning platform helps me prepare better for the MLP exam.	2.15 (0.76)	0.64
	The MLP e-learning platform helps me think through medical problems.	2.11 (0.59)	0.54
	Using the MLP e-learning platform has helped me to accomplish my learning tasks more efficiently.	2.25 (0.62)	0.66
	Using the MLP e-learning platform has made my learning activities become much easier than without.	2.14 (0.66)	0.63
	My learning performance [is] enhanced since using the e-learning platform.	2.04 (0.62)	0.63
	I find the MLP e-learning platform useful for my studies.	1.92 (0.55)	0.59
	The MLP e-learning platform saves me money.	1.96 (0.66)	0.62
	The e-learning materials improve my clinical performance.	1.93 (0.54)	0.60
	I feel that the e-learning platform has a direct positive impact on being an MLP practitioner.	1.83 (0.53)	0.56
	The MLP e-learning platform helps me to treat patients better.	1.82 (0.48)	0.53
	The MLP e-learning platform saves me time.	1.90 (0.56)	0.62

^a^MLP: medical licentiate practitioner.

^b^*r*: corrected item-total correlations.

^c^IT: information technology.

### Objectives

The primary objective of the study was to evaluate the technology acceptance and IS success of the e-platform.

### Statistical Methods

All data were cleaned in a systematic screening for completeness, plausibility, and consistency. Potential inconsistencies were resolved by checking against original data forms. Results were transferred into a spreadsheet and data were then analyzed with RStudio Desktop (open-source license version 1.1.463) [19].

For questionnaire items, we calculated the mean, standard deviation, and correlation coefficient *r* to see how well aligned questions were to the respective categories. Likert scale data were interpreted as interval scales [20], and data from various study participants were regarded as independent as we assumed that the behavior of one participant did not influence the behavior of another. Correlations of variables were explored with Kendall tau correlation coefficient as an estimator of correlation in the population.

**Ethical Considerations**

We communicated the purpose of this research to study participants and explained the study design and participation requirements. Before taking part in this study, all study participants agreed to and signed an informed consent explaining the study scope and purpose, and their right to withdraw at any point. Voluntary study participation was emphasized to be voluntary and assessments were solely part of this study. Furthermore, it was emphasized that the usage of the e-platform is tracked. The Biomedical Research Ethics Committee of the University of Zambia and the ethical committee of the University Hospital Heidelberg, Germany approved the study protocol.

## Results

### Demographics

Overall, the student population was predominantly male (58/74; 78%) with female students in the minority (16/74; 22%). The predominant age group was between 30 to 40 years (42/74; 57%; see Table 2). Most students had had more than five years of medical experience with an average of 10 years of working experience. Medical lecturers in the MLP were also predominantly male (10/14; 71%) with an average work experience of approximately 17 years and an average of 10 years of teaching experience.

Female students’ experience with technology was generally low, whereas males reported a predominantly midrange of technology experience (see Multimedia Appendix 3). Demographic details for age groups and medical and technology experience by gender are presented in Table 2.

Owing to a high number of missing data values, data from 9 MLP students were excluded from further analysis.

### Technology Acceptance

With regard to technology acceptance, the highest rated categories by MLP students were *Behavioral Intention* (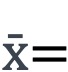
1.69; SD 0.58; *r*=0.67) and *Attitude* (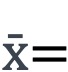
1.72 SD=0.59; *r*=0.70), whereas *Subjective Norm* (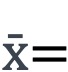
1.92; SD=0.61; *r*=0.78) and *Self-Efficacy* (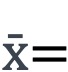
1.86; SD=0.69; *r*=0.65) were the categories with the lowest acceptance among students, followed by the categories of perceived usefulness (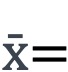
=1.85; SD=0.61; r=0.60) and perceived ease of use (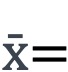
=1.82; SD=0.61; *r*=0.61; see Table 3). The individual items with the highest agreement among MLP students were as follows: “For my future job, it is necessary to know how to use a computer” (item 21; 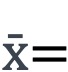
1.59; SD=0.55; *r*=0.77), “I am positive towards using a tablet for medical learning” (item 18; 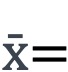
1.60; SD=0.57; *r*=0.59), and “In my job, using a tablet is important” (item 13; 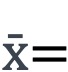
1.61; SD=0.52; *r*=0.64). The least agreement MLP students had were on the following items: “Using a tablet computer is compatible with all aspects of my studies” (item 10; 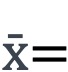
2.04; SD=0.78; *r*=0.64), “E-learning makes it easier to study course content” (item 9; 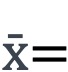
1.96; SD=0.62; *r*=0.49), and “Learning how to use the MLP e-learning platform is easy for me” (item 2; 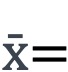
1.96; SD=0.60; *r*=0.57).

### Information System Success

For the IS success questionnaire, the category of Information Quality received the overall best ratings from MLP students (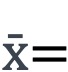
1.99; SD=0.80; *r*=0.57) followed by Net Benefits (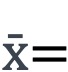
2.00; SD=0.61; *r*=0.60) that contrasted with System Use with the lowest rating (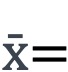
2.47; SD=0.84; *r*=0.69) and Service Quality (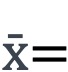
2.29; SD=0.86; *r*=0.52) with the second lowest rating (see Tables 3 and 4). The specific items with the highest agreement among MLP students were all in the category of information quality, “Materials available on the MLP e-learning platform are useful to me” (item 3; 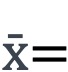
1.58; SD=0.56; *r*=0.49), “The MLP e-learning provides information relevant to MLP medical practice” (item 6; 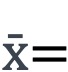
1.63; SD=0.55; *r*=0.56), and “Materials on the MLP e-learning platform increase my quality of clinical care” (item 7; 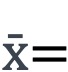
1.74; SD=0.51; *r*=0.57). Items rated the lowest by MLP students were in the lowest-rated categories of system use and service quality, “I use the MLP e-learning platform daily several times” (item 32; 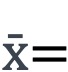
2.61; SD=0.90; *r*=0.76), “I depend upon the MLP e-learning platform” (item 17; 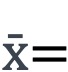
2.54; SD=0.92; *r*=0.38), and “The MLP e-learning platform provides rapid services” (item 33; 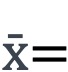
2.53; SD=0.82; *r*=0.72).

The Kendall tau variable correlation coefficients are shown in Tables 5 and 6 for the IS success model and TAM questionnaires, respectively. The bivariate relationship indicated that all of the variables (questionnaire items) were significantly correlated (correlations<0.05; see Tables 5 and 6).

For medical lecturers, the categories *Perceived enjoyment* (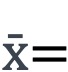
1.34; SD=0.48; *r*=0.73) and *Perceived ease of use* (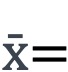
1.86; SD=1.05; *r*=0.48) were rated the highest, whereas *Behavioral intention* (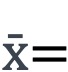
2.14; SD=0.83; *r*=0.54) and *Self-efficacy* (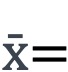
2.11; SD=1.19; *r*=0.72) were perceived as the categories with the least agreement (see Table 7). The Kendall tau variable correlation coefficients for the TAM questionnaire are shown in Table 8. Furthermore, the questions asked in addition to the TAM disclosed that 2 medical teachers had never used the e-platform (never n=2; often n=3; once n=1; several times n=1; frequently n=1) and 5 did not know how to access it. Overall, 50% (7/14) of medical lecturers stated that they had contributed to the e-platform with content or other e-learning–based activities, such as virtual patients (n=7). Correlation coefficients (Kendall ; nonparametric correlation) of the medical lecturers’ technology acceptance items indicated a correlation for most variables (see Table 8). High correlation significance (*P*<.01) was shown between the items perceived usefulness and attitude, perceived ease of use and self-efficacy, and perceived ease of use and perceived usefulness.

**Table 5:** Correlations (Kendall τ) of information system success model of the Zambian medical licentiate practitioner student questionnaire.

Categories of information system success model	Service quality (ServQ)	System quality (SQ)	User satisfaction (US)	System use (SU)	Net benefits (NB)
Information quality (InfQ)	0.23^a^	0.36^b^	0.41^b^	0.30^b^	0.40^b^
ServQ	—^c^	0.35^b^	0.25^a^	0.26^a^	0.22^a^
SQ	—	—	0.49^b^	0.31^b^	0.37^b^
US	—	—	—	0.40^b^	0.61^b^
SU	—	—	—	—	0.46^b^
NB	—	—	—	—	—

^a^*P*<.05.

^b^*P*<.01.

^c^Not applicable.

**Table 6:** Correlations (Kendall τ) of adapted technology acceptance model of the Zambian medical licentiate practitioner student questionnaire.

Categories of technology acceptance model	Perceived usefulness (PU)	Attitude (AT)	Behavioral intention (BI)	Self-efficacy (SE)	Subjective norm (SN)	Information system success (IS)
Perceived ease of use	0.48^a^	0.46^a^	0.48^a^	0.31^b^	0.31^b^	0.36^a^
PU	—^c^	0.53^a^	0.41^a^	0.52^a^	0.47^a^	0.44^a^
AT	—	—	0.59^a^	0.44^a^	0.39^a^	0.35^a^
BI	—	—	—	0.47^a^	0.37^a^	0.22^b^
SE	—	—	—	—	0.46^a^	0.36^a^
SN	—	—	—	—	—	0.38^a^
IS	—	—	—	—	—	—

^a^*P*<.01.

^b^*P*<.05.

^c^Not applicable.

**Table 7:** Questionnaire results of technology acceptance model questionnaires of medical licentiate practitioner lecturers.

Questionnaire items of technology acceptance component: MLP^a^ medical lecturers		Mean (SD)	*r* ^b^
**Attitude**		1.90 (0.80)	0.39
	I intend to use the MLP e-learning platform to assist my medical teaching.	1.50 (0.67)	0.37
	I am positive towards the MLP e-learning platform.	1.92 (0.79)	0.40
	I believe that working with tablets is for young people only.	1.25 (0.45)	0.42
	I believe that working with computers is for young people only.	1.25 (0.45)	0.42
	Most MLP medical lecturers bring a positive attitude towards e-learning.	2.58 (0.67)	0.50
	I want to dedicate more effort to support the MLP e-learning platform.	2.08 (1.08)	0.37
	The MLP e-learning platform can be successfully established for the MLP program.	2.08 (0.67)	0.36
	I think the MLP e-learning platform can be established as a permanent part of the MLP program.	2.08 (0.67)	0.36
	E-learning is a good tool for the MLP program at CCHS.	1.83 (0.72)	0.36
	I think the MLP e-learning platform is important for MLP students.	2.08 (0.79)	0.38
**Behavioral intention**		2.14 (0.83)	0.54
	I want to upload content myself on the MLP e-learning platform.	2.36 (1.03)	0.42
	My input is vital for the success of the MLP e-learning platform.	2.18 (0.75)	0.41
	I actively want to contribute to the MLP e-learning platform.	1.91 (0.54)	0.41
	I think other MLP medical lecturers want to regularly contribute to the MLP e-learning platform.	2.45 (0.93)	0.39
	I want to regularly contribute content to the MLP e-learning platform.	2.09 (1.14)	0.50
	I am willing to take ownership for the MLP e-learning platform.	2.09 (0.83)	0.37
	The MLP e-learning platform is only temporary.	2.27 (0.79)	0.37
	The MLP e-learning platform can be sustained long-term at CCHS.	2.27 (0.79)	0.37
	I think other MLP medical lecturers want the MLP e-learning platform to be successful.	1.91 (0.83)	0.36
	I want the MLP e-learning platform to be successful.	1.82 (0.60)	0.35
**Perceived ease of use**		1.86 (1.05)	0.48
	Working with computers is easy for me.	1.36 (0.50)	0.07
	I am using computers as a tool for teaching.	1.36 (0.50)	0.04
	Learning how to use the MLP e-learning platform is easy for me.	2.36 (0.92)	0.16
	I need training to actively contribute to the MLP e-learning platform.	2.36 (1.50)	0.22
**Perceived usefulness**		2.00 (0.84)	0.45
	The use of The MLP e-learning platform is better as compared to textbook learning.	2.45 (0.82)	0.45
	Overall, the use of The MLP e-learning platform is more time-demanding than traditional teaching methods.	2.00 (1.26)	0.49
	The MLP e-learning platform provides quality medical materials for MLP students.	2.00 (0.77)	0.44
	The MLP e-learning platform is a useful tool for the MLP program.	1.82 (0.75)	0.43
	I think e-learning platform improves my productivity as a medical lecturer.	2.09 (1.04)	0.45
	I think e-learning improves my effectiveness as a medical lecturer.	1.82 (0.98)	0.43
	I think e-learning improves my teaching performance.	1.82 (0.98)	0.43
	I believe using e-learning is helpful for my teaching.	1.64 (0.81)	0.44
	E-learning makes it easier to teach medical courses and their content.	1.91 (0.70)	0.43
	I believe working with computers makes a person more productive at their job.	1.73 (1.01)	0.46
	I think the MLP e-learning helps students to be better MLPs.	2.27 (0.65)	0.45
	I think that the MLP e-learning can improve quality of learning for MLP students.	1.73 (0.65)	0.44
	I think the MLP e-learning platform needs too much effort to use it for teaching.	2.27 (0.90)	0.51
	I think the MLP e-learning platform improves the effectiveness of medical teaching.	1.91 (0.70)	0.44
	I think the MLP e-learning platform can save me effort.	1.82 (0.75)	0.47
	Overall, I think the MLP e-learning platform can save me time.	1.91 (0.70)	0.45
	The MLP e-learning platform improves medical performance of MLP students as MLP practitioners.	2.45 (0.82)	0.46
	The MLP e-learning platform improves the clinical performance of MLP students.	2.18 (0.75)	0.44
	Tablets are useful as learning devices for MLP students.	2.00 (0.77)	0.44
	The MLP e-learning platform improves learning outcomes of MLP students.	2.18 (0.87)	0.44

^a^MLP: medical licentiate practitioner.

^b^*r*: item-total correlation.

^c^Reverse scoring of Likert items applied.

**Table 8:** Correlation (Kendall τ) of adapted technology acceptance model of Zambian medical licentiate practitioner lecturer questionnaire.

Categories of technology acceptance model	Perceived usefulness (PU)	Attitude (AT)	Behavioral intention (BI)	Self-efficacy (SE)
Perceived ease of use	0.40^a^	0.53^b^	-0.02	0.62^b^
PU	—^c^	0.75^d^	0.44^a^	0.60^b^
AT	—^c^	—^c^	0.32	0.53^b^
BI	—^c^	—^c^	—^c^	0.11
SE	—^c^	—^c^	—^c^	—^c^

^a^*P*<.10.

^b^*P*<.05.

^c^Not applicable.

^d^*P*<.001.

## Discussion

### Principal Findings

Overall, the questionnaires based on the TAM and IS success model indicated a positive reception of the e-platform and its usage with the offline-based tablets by MLP students and medical lecturers and identified strengths and shortcomings in the tablets and MLP e-platform. The questionnaires’ results elicited an overall acceptant attitude toward the e-platform and agreement with the technological environment of e-learning as a mode of learning and teaching by both, MLP students and medical lecturers.

The student study participants were quite heterogeneous in age, medical experience, and technological experience. The youngest student was aged 23 years and the oldest was 54 years. Some students had only 1 year of medical experience whereas others had accumulated over 28 years of medical experience. The same was applied to technological experience, as some students’ exposure to the MLP e-platform was the first time they had ever had a chance to experience continuous technology use for learning and accessing knowledge. Others, *digital natives* who had grown up with technologies, came into the MLP program equipped with 2 mobile devices and a laptop as an inherent part of their daily lives. Males seemed more confident in rating their self-perceived technology experience than females, who seemed more conservative in their self-assessment. Potentially, this finding may be the result of the prevailing gender-based stereotypes holding females back, as technology usage may not fit the stereotype of expectations for African females. In general, female students may be faced with “a subconscious or implicit bias against women scientists in higher education settings” as “stereotypes emerge early and continue to be salient throughout the lifespan” [21]. The implicit bias against women, which is unfortunately still prevalent, should be taken up and actively approached in the MLP e-platform’s implementation strategy. One study found, “for instance, college students were more likely to rate the same conference abstracts as lower in scientific quality if the author’s name was female instead of male, particularly if these topics had traditional masculine themes” [21]. Another study described a “moderating effect of gender difference in the adoption of multimedia technology for learning,” whereby males, “believe the technology can improve their performance when they find a fit between task and technology, and this belief would lead to their adoption of the technology” [22]. The study recommended (only for males) “that educators can better promote male students' adoption of multimedia technology by demonstrating how appropriate the multimedia technology given is for their learning” [22].

Age too can be a discriminative factor in technology acceptance and should be taken into consideration particularly for heterogeneous cohorts like the MLP students. “More attention should be paid to their support [elder MLP students] in the form of training, as well as meeting their unique personal needs” [23]. Overall, elder MLP students were more satisfied with the MLP e-platform than younger MLP students. The higher satisfaction may have been rooted in older students’ limited prior exposure to technology and consequently lower expectation levels. Thus, elder MLP students may have perceived the MLP e-platform as more satisfying than their younger colleagues.

In particular, MLP e-platform information quality and net benefits were perceived to be the most appealing characteristics based on the IS success model. A closer look at the individual questions of those 2 categories, however, shows that the ratings were more differentiated since students rated available materials as clear and useful, but questions about materials’ quality and sufficiency received the lowest overall scores (information quality). The quality and quantity of contents was perceived as a hindrance which may have been rooted in the short implementation period of the MLP e-platform in general which resulted in limited and rather static learning content that did not entirely reflect the MLP curriculum. Contents were initially composed only of lecture notes and adapted materials from a Malawian e-learning platform [24,25].

To this end, adopting a social media style for content creation and curation to allow for student-made, peer-reviewed content based on fellow student ratings, mixed with methods such as microlearning or learning nuggets, could potentially leverage the practicality of mobile devices (eg, tablets), and could be a significant next step for higher acceptance learning [26]. Curating available Web-based content may be of benefit to keep contents up to date while at the same time decreasing efforts to develop own learning content which constitutes a current major bottleneck—resource-intense and manual content creation. The MLP e-platform may then act also as a central hub for grouping and sharing reliable and appropriate information from the internet [27].

Students underlined with their high acceptance of the statements “For my future job, it is necessary to know how to use a computer,” “I am positive towards using a tablet for medical learning,” and “In my job, using a tablet is important” that they are willing and know the importance to make use of technologies, such as tablets, for their clinical work. Consequently, the categories of perceived usefulness denoting the relevance of the MLP e-platform toward job performance and perceived ease of use also had high agreement among MLP students. Similarly, for e-platform net benefits, the students found it useful in general, especially using the tablet in a clinical context for treating patients, but the e-platform did not support students well for their exams or help them achieve their learning tasks more efficiently. The subjective norm, *a person's perception that most people who are important to him think he should or should not perform the behavior in question* [28], was not rated as a strong factor compared with other questionnaire categories nor was e-platform self-efficacy, *people's judgments of their capabilities to perform a given task* [29]. The students’ low ratings for the subjective norm can be attributed to the e-platform still being a relatively new mode for the MLP. The MLP students did not find the tablet compatible with all aspects of their studies, nor did they perceive that the e-platform made studying easier. This shows a need for adaptation of the e-platform to better suit its users and enhance the overall ease of use. During the SY, tablet failure impacted the reliable usage of the offline e-platform (service quality). Most tablets failed either because of poor operating system upgrades or hardware fragility (for a more detailed description see the study by Barteit et al [9]). Furthermore, for some tablets, the battery life span reduced rapidly limiting its usage. Service quality and the quality of the materials, in turn, may have had an impact on the students’ low ratings for self-efficacy, which has been shown to have *a direct and powerful effect on actual use over and above user intention* [29].

Medical lecturers showed a general acceptance of technology for learning and teaching within the MLP program as the highest ratings for the questionnaires were given for perceived ease of use and perceived usefulness of the e-platform. However, most medical lecturers rated their colleagues’ attitude toward the MLP e-platform as not very positive, which indicates that many did not count on digitized methods to be a part of their teaching and potentially do not see these methods as valid instruments. Their perception toward the e-platform to improve MLP student medical performance received low agreement scores, which further underlines skepticism toward e-learning as a teaching method. In addition, medical lecturers conveyed low confidence toward using the e-platform for medical teaching in combination with a potentially conservative preference for textbook-based learning and lecture-style teaching as a core teaching method. The item, *The use of the ML e-learning platform is better as compared to textbook learning*, received one of the lowest ratings of all questions, and the categories of behavioral intention and self-efficacy of the e-platform both received very low scores indicating low agreement among medical lecturers. These low ratings further manifest a certain reluctance to employ the e-platform for teaching. Potentially, a bias was rooted in social desirability which influenced MLP lecturers’ answers to the questionnaire [30] as they knew that they were expected to show a certain openness to technologies. Thus, the results of this evaluation suggest a low medical lecturer uptake of the e-platform, which potentially resulted in limited learning materials and restricted integration of the e-platform in day-to-day teaching and training [9,31]. One MLP medical lecturer suggested increasing the allowance for *site consultants so as to motivate them* to make use of the e-platform. In fact, an increase in lecturer payment in Mexico showed no benefit, as it did not translate into an impact on student learning nor did it hold as a strategy for better teacher engagement in the digital learning and teaching process [32]. A more promising approach seems to be to consider the transformation of the *teaching-learning process* that entails a global approval and acceptance of teachers and the administration of digital technologies. Technology cannot fix outdated processes and substitute for well-qualified, motivated teachers. Thus, it may be of benefit to further educate and support teachers to use digital technologies for teaching and training [31], including content creation and e-learning didactics, such as flipped classrooms, adaptive learning [33], video-making with low-entry technologies such as smartphones, short mobile learning sessions (microlearning for on-the-job learning), and fostering technology-based social learning activities such as discussion and learning groups. An increased institutional presence may also improve lecturer approval and acceptance, such as an e-learning lab, which may take the physical shape of a dedicated part of the library with a few computers, where medical lecturers can seek technical support for making use of the e-platform or to prepare e-platform contents. Attitude has been identified as an important factor contributing to technology acceptance and may well change over time when more technological experience is gained [34]. Training teachers in digital literacy is not only of benefit for catering for the *teaching-learning process* but also may provide the cobenefit of preparing them to handle and embrace technologies in the medical field, which are pervasive and rapidly increasing as the digitization of medicine progresses [35,36].

Overall, medical lecturers did show a readiness and acceptance toward technology, since the majority negated that technologies were only for young people and agreed that they intended to use the e-platform to assist their medical teaching and that the e-platform could be helpful for their teaching.

### Limitations

The limitations of the study are as follows:

- Ensuring questions are clear and not misleading: (1) Getting respondents to answer questions thoughtfully and honestly and (2) obtaining a sufficient number of completed questionnaires to enable meaningful analyses [37].
- No comparison group: All study participants were subject to the same intervention, so it was difficult to attribute the change in outcome to the introduction of the program with any certainty.

### Conclusions

MLP students and medical lecturers accepted the e-platform as a method for medical teaching and learning and the evaluation of the MLP e-platform with its offline tablet-based component, which proved to be a feasible approach for teaching and learning within the low-resource environment at the CCHS in Zambia. This overall positive acceptance toward the e-platform constitutes a fertile base to scale up and implement the e-platform as a serious learning and teaching methodology within the blended learning strategy for the MLP program and beyond. Main shortcomings comprise the low uptake of the e-platform in everyday teaching and the scope and level of interactivity of the e-platform contents. The e-platform has the potential to be a cornerstone in the expansion of scaling up the training of urgently needed medical licentiates in Zambia, but it requires a profound transformation of the teaching and learning process that at its core is manifested in the curriculum and ongoing technology training for medical lecturers. From a technological standpoint, the tablet-based e-platform is a flexible tool that allows for electronic assessment, skill-based learning sessions, a digital logbook for the MLP, and an enhanced clinical decision-support system that could be incorporated in the continuous medical training for MLP graduates in rural health facilities. With increasingly affordable and viable technologies being available, the e-platform may potentially become a game changer in future medical education, especially in a low-resource context such as Zambia.

## Appendix

Multimedia Appendix 1 Questionnaire for medical licentiate practitioner students comprising demographic data and prior technological experience.

Multimedia Appendix 2 CONSORT eHealth Checklist.

Multimedia Appendix 3 Level of technology experience amongst medical licentiate practitioner (MLP) student study population by gender.

## Abbreviations

CCHS: Chainama College of Health Sciences
e-learning: electronic learning
e-platform: electronic health platform
IS: information system
ISSM: information system success model
MLP: medical licentiate practitioner
SY: study year
TAM: technology acceptance model
