# Supplementary material for: Technology Acceptance and Information System Success of a Mobile Electronic Platform for Nonphysician Clinical Students in Zambia: Prospective, Nonrandomized Intervention Study
Source: J Med Internet Res. 2019 Oct 9;21(10):e14748. doi: 10.2196/14748 (PMC6914109; doi:10.2196/14748)
Supplement: Multimedia Appendix 1 [file jmir_v21i10e14748_app1.pdf]

Appendix 1: Questionnaire for medical licentiate practitioner students comprising demographic data and prior technological experience.

ELSE KRÖNER-FRESENIUS-STIFTUNG
Forschung fördern. Menschen helfen.

SOLIDAR MED

Participant Details

We will not be sharing information about you to anyone outside of the research team. The information that we collect from this research project will be kept private. Only the researchers will have access to this information and information is locked up. Any information collected will not be shared with or given to anyone except the researchers involved in the research.

Participant Details	
First Name:	
Last Name:	
Date:	
Place:	
DOB:	
Gender:	
Marital Status	
Which study year?	❑ 2nd ❑ 3rd ❑ 4th ❑ Bridging
What did you study before joining the ML programme?	
Which year did you graduate as Clinical Officer?	
How many years of uninterrupted medical practise do you have?	
Do you have a technical device available for your own usage?	❑ Laptop ❑ Workstation<br>❑ Tablet ❑ No device<br>❑ Else:
➢ If yes, do you use your technical device for medical learning within the ML programme?	❑ Yes ❑ No ❑ Else:
➢ If yes, how often do you use the technical device available to you for learning for the ML programme?	❑ A few times per month ❑ Every other week<br>❑ Once a week ❑ Every other day ❑ Daily

How would you, as objectively as possible, see yourself using technical devices like computers, tablets, smartphones?	❑ With great ease<br>❑ Comfortable in using<br>❑ Okay<br>❑ Not really comfortable<br>❑ I hardly can use them
How would you, as objectively as possible, see yourself navigating through the Internet?	❑ With great ease<br>❑ Comfortable in using<br>❑ Okay<br>❑ Not really comfortable<br>❑ I hardly can use a computer
Have you participated in an e-learning class or participated in an online learning course?	❑ Yes ❑ No ❑ Else:
➢ If yes, what kind of e-learning class was it?	
➢ If yes, how would you describe your experience with the e-learning (positive, beneficial, not helpful etc.)?	
Do you think e-learning could be useful to you within the ML program?	❑ Yes ❑ No ❑ Else:
What contents and materials would you like to find on the ML e-learning platform?	
