# Supplementary figures and images for: Technology Acceptance and Information System Success of a Mobile Electronic Platform for Nonphysician Clinical Students in Zambia: Prospective, Nonrandomized Intervention Study
Source: J Med Internet Res. 2019 Oct 9;21(10):e14748. doi: 10.2196/14748 (PMC6914109; doi:10.2196/14748)

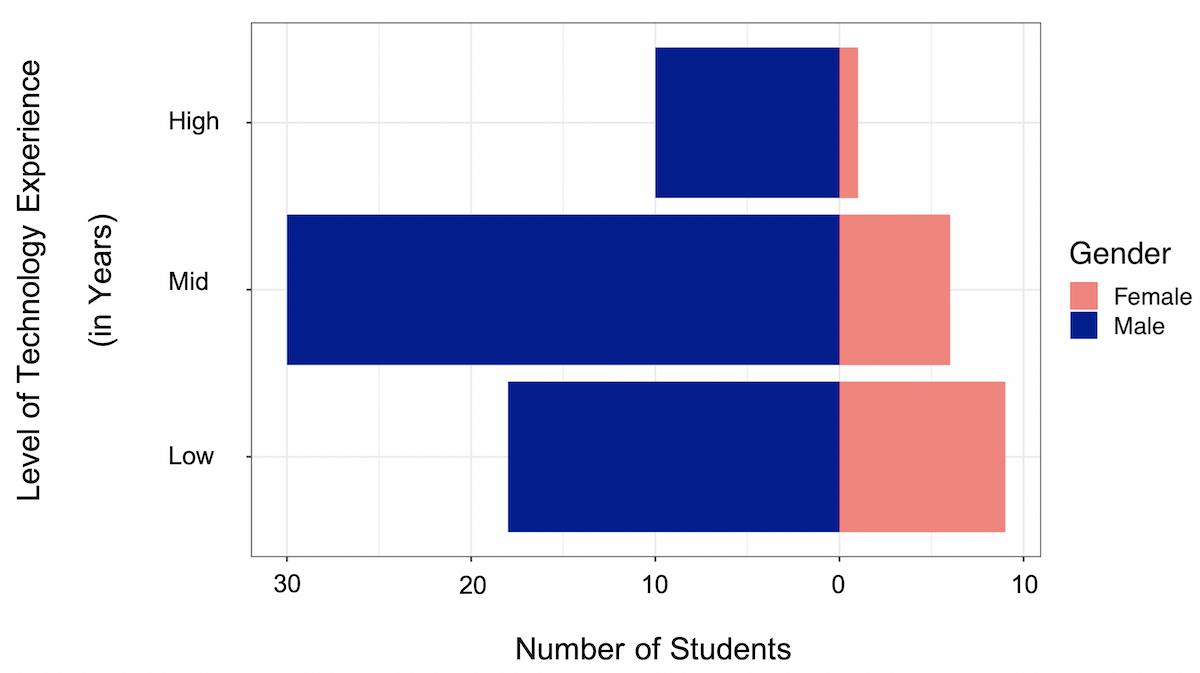

Supplement: Multimedia Appendix 3 [file jmir_v21i10e14748_app3.png]
